# An Exploratory Single-Arm Clinical Trial on Eucommia Ulmoides Leaf Extract Effects on Blood Pressure, Oxidative Stress, and Atrial Natriuretic Peptide (ANP) in Individuals With and Without Chronic Kidney Disease

**DOI:** 10.1155/ijne/5598055

**Published:** 2025-06-19

**Authors:** Hiroshi Satonaka, Shohei Yokoyama, Akira Ishimitsu, Chisato Takahashi, Tatemitsu Rai, Daisuke Nagata, Toshihiko Ishimitsu, Akihiro Tojo

**Affiliations:** ^1^Division of Nephrology, Department of Internal Medicine, Jichi Medical University, 3311-1, Yakushi-ji, Shimotsuke-shi, Tochigi 329-0498, Japan; ^2^Department of Nephrology and Hypertension, Dokkyo Medical University, 880, Kitakobayashi, Mibu, Shimotsuga-gun, Tochigi 321-0293, Japan

## Abstract

Eucommia ulmoides (Tochu) has been shown to possess a variety of beneficial effects on profiles affecting the onset of lifestyle- or aging-related diseases, such as hypertension, diabetes, dyslipidemia, or obesity. This exploratory single-arm clinical study was conducted on a total of 17 participants including those with mild chronic kidney disease (CKD) (*n* = 9), who were administered a tablet product containing Tochu leaf extract for a short period (median: 33 days), to investigate its effects on blood pressure or related clinical markers. Mean systolic blood pressure (SBP) of all the participants significantly decreased from 128.3 ± 12.3 mmHg at the start to 123.8 ± 10.2 mmHg at the end of the administration (*p* < 0.05). Analysis of CKD patients alone, however, revealed that SBP, to a greater extent, decreased from 130.7 ± 12.9 mmHg to 121.2 ± 10.7 mmHg (*p* < 0.01), while the change in non-CKD patients was not significant. Furthermore, SBP decrease in CKD patients with hypertension (*n* = 7) alone was also significant and comparable. Mean blood oxidative stress index of all participants was decreased from 300.2 ± 76.7 U.CARR to 285.9 ± 63.0 U.CARR (*p* < 0.05), while median atrial natriuretic peptide (ANP) of all the participants was increased from 8.1 (5.0–9.6) pg/mL to 8.8 (5.8–12.1) pg/mL (*p* < 0.05). Our findings suggested that Tochu-derived components may have potential therapeutic benefit at earlier stages in CKD, which could fill the gaps in currently underserved opportunities for prevention or intervention.

**Trial Registration:** UMIN Clinical Trials Registry: UMIN000050727

## 1. Introduction

Alongside the drug treatment, use of foods and food-derived ingredients can be effective in preventing or treating aging- or lifestyle-related diseases. It is stated in International Society of Hypertension Global Hypertension Practice Guidelines (2020) that consuming beverages, including coffee, green, or black tea, in moderation may be beneficial [1].

Eucommia ulmoides (Tochu) bark has historically been used as herbal medicine, while the leaves have been used as food materials [2]. It can positively affect blood pressure [3], hyperlipidemia [4], oxidative stress [5, 6], hyperglycemia [7], and obesity [8].

Numerous compounds, including iridoids, flavonoids, phenols, and triterpenoids, have been isolated and identified from glycosides extracted from Tochu leaves [2]. Geniposidic acid (GEA), an iridoid glycoside, has been shown as one of Tochu's important components of pharmacological relevance. Tochu leaf glycoside, which contains GEA, has a blood pressure-lowering effect as demonstrated by preclinical studies in spontaneously hypertensive rats (SHRs) [9]. GEA has been shown to reduce vascular resistance via relaxation of vascular smooth muscle with the involvement of muscarinic acetylcholine receptors [10]. There have also been reports on the mechanism of nitric oxide (NO)-mediated improvement in vascular endothelial function [11]. On the other hand, Nakamura has shown NO-independent and atrial natriuretic peptide (ANP)-mediated antihypertensive effect of GEA in SHRs, which was presumably mediated by glucagon-like peptide-1 receptor (GLP-1R) with GEA functioning as its agonist [12]. This seemed in agreement with previously demonstrated mechanisms whereby liraglutide promoted ANP secretion from cardiomyocytes via ligation to GLP-1R as agonist [13].

A drink product containing Tochu leaf glycosides has been approved to be labeled as a “food suitable for people with higher blood pressure” under the framework of Food for Specified Health Uses (FOSHU) formulated by the Japanese Ministry of Health and Welfare. The GEA content in this product, determined by high-performance liquid chromatography (HPLC), was specified as an indicator of its function as a FOSHU. In a clinical study which was randomized and placebo controlled, the drink product containing 85 mg of GEA per bottle was administered once a day to people with “high normal” blood pressure, which was defined as with either systolic blood pressure (SBP) 130–139 mmHg or diastolic blood pressure (DBP) 85–89 mmHg, and without overt hypertension (SBP ≧ 140 mmHg or DBP ≧ 90 mmHg) [14]. Vascular endothelial function according to flow-mediated dilatation (FMD) was improved by this treatment in 4–8 weeks, accompanied by a decrease in blood pressure.

The number of patients with chronic kidney disease (CKD) is on the rise, and the aging of society and the increasing prevalence of diabetes and hypertension are part of the important factors [15]. Hypertension causes nephrosclerosis and is thus an important etiology of CKD, and of further importance, therapeutic intervention against high blood pressure has been shown to be generally inhibitory against CKD progression [16]. Effects of Tochu on renal pathophysiology have only been investigated in a limited number of preclinical studies. In a study with Dahl salt-sensitive (DS) rats, Tochu tea extract and GEA suppressed NADPH oxidase, increased eNOS, and improved blood pressure and renal hemodynamics [17]. In our present study, which may be the first to clinically examine effects of Tochu in the context of CKD, a tablet product containing Tochu leaf extract was administered to participants including those with mild CKD, and its effects on blood pressure and related clinical indices were explored.

## 2. Methods

### 2.1. Study Design and Participants

This study was conducted as an exploratory single-arm intervention trial at the outpatient clinic of Department of Nephrology and Hypertension, Dokkyo Medical University. According to the protocol selection criteria, the participants were required to be patients with CKD of stages G1–G3a, namely, with eGFR of 45–60 mL/min/1.73 m^2^ or with positive proteinuria (≧ 0.15 g/gCr), based on Evidence-Based Clinical Practice Guideline for Chronic Kidney Disease 2023 by Japanese Society of Nephrology [18]. According to the CKD staging in this guideline, either albuminuria or proteinuria categories can be adopted, where A2 (either 0.15–0.49 g/gCr in proteinuria or 30–299 mg/gCr in albuminuria) and A3 (≧ 0.50 g/gCr in proteinuria or ≧ 300 mg/gCr in albuminuria) categories are regarded as positive while A1 (< 0.15 g/gCr in proteinuria or < 30 mg/gCr in albuminuria) is negative. The participants were also to be 20 years of age or older and to have given their written consent to participate in the study. They were excluded from participation if they were either with a malignant disease, with cognitive dysfunction or were, otherwise, judged by the physician to be unsuitable as study participants due to comorbidities.

### 2.2. Intervention

As the food product to be tested in this study, a tablet formulation which was registered in the category of “Food with Functional Claims,” with number D160 to Consumer Affairs Agency of Japan, was used. For the manufacturing of the daily dose (9 tablets) of this product, which contained 85 mg of GEA, 1416.7 mg of Tochu leaf extract was used. Other nutritional components per 9 tablets included protein < 0.13 g, lipids 0.017–0.17 g, carbohydrates 2.4 g, NaCl < 0.0046 g, and calcium 4.8–48 mg, totaling 10.5 kcal in energy, as based on the information for the product registration. During the study treatment period, recommended daily dose (9 tables) was administered to all the participants for an intended period of 8–12 weeks, depending on the necessities of clinic visit schedule. During the study period, medications other than the study tablet were continued, and there were no specific instructions given otherwise to make any changes to daily habits such as diet, including intaking of other tea beverages.

### 2.3. Outcomes

At the start and at end of the treatment period, blood and urinary samples were collected for research as well as for routine clinical purposes. Diacron-reactive oxygen metabolites (d-ROMs), a circulating oxidative stress marker, and the biological antioxidant potential (BAP) were measured using frozen plasma samples with the use of an analytical system following the recommended protocol (Wismerll, Tokyo, Japan). As a metabolic marker, adiponectin, an adipokine involved in glucose and fatty acid regulation, was measured. As inflammatory markers, malondialdehyde-modified low-density lipoprotein (MDA-LDL), which is a representative oxidized LDL, as well as CRP was measured. As an index of vascular endothelial function, peripheral blood vascular endothelial progenitor cell (EPC) levels were measured, expressed as percentages of CD133- and Flk-1-double-positive cells in mononuclear cell fraction, by flow cytometry as previously described [19]. Plasma ANP, a potential blood pressure modulating factor as described above, was measured by ELISA. Blood pressures at the outpatient clinic were measured at rest in sedentary position.

### 2.4. Ethics

The study protocol obtained ethical approval by the University institutional clinical research review board on September 14, 2021, with No. R-50-1. Study participants were enrolled in 2022 and 2023. This study complies with the Declaration of Helsinki and the Japanese Ethical Guidelines for Medical and Biological Research Involving Human Subjects.

### 2.5. Statistical Analysis

Values are presented as the mean ± standard deviation or median (interquartile range) for continuous variables and percentage for categorical variables. Values of different groups were compared by the unpaired *t*-test or Wilcoxon rank-sum test, while values of the same group at different time points were compared by the paired *t*-test or Wilcoxon signed-rank test. Some of the ANP values below the lowest detection limit (5 pg/mL) were assigned as 5 pg/mL in Wilcoxon's signed rank test. *p* values < 0.05 were regarded as statistically significant. JMP (Version 17.2.0) was used for statistical analyses.

## 3. Results

A total of 17 participants were included in the study. Nine had CKD (with eGFR < 60 mL/min/1.73 m^2^ or urinary protein ≧ 0.15 g/gCr). The other eight, with eGFR ≧ 60 mL/min/1.73 m^2^ and urinary protein < 0.15 g/gCr, did not have CKD by definition. This turned out to be a deviation from the protocol, to be exact, which happened largely due to issues relating to study subject recruitment. The actual intention of the study, however, had originally been to conduct a preliminary exploration in a population with relatively early CKD, which almost had healthy individuals in scope as well.

Average age of all 17 participants was 59.3 ± 16.2 years (Table 1). Fourteen (82%) were male. Estimated GFR of all the participants was 67.6 ± 15.7 mL/min/1.73 m^2^, and the eGFR of CKD patients only was 60.5 ± 17.6 mL/min/1.73 m^2^. Duration of the study tablet administration varied according to the circumstances of hospital visit schedule of individual participants. As the result, actual duration of all the participants was median 33 days, shorter than what was initially intended in the protocol. Participants with CKD were treated for median 28 days, significantly shorter than those without CKD who were treated for median 35 days.

As to major background diseases in the participants with CKD, seven had hypertension, four had chronic glomerular nephritis, and three had diabetes mellitus (Table 2). In some patients, there was overlap of these diseases. There were no participants who had clinically diagnosed heart failure or arrhythmia, though there was one CKD patient who had history of percutaneous coronary intervention. Among the baseline prescribed drugs with potential implication for CKD treatment, antihypertensive drugs included CCB, ARB, thiazide, beta-blocker, and MRA, often with overlap of multiple drug classes in each CKD patient (Tables 1 and 2). Otherwise, frequency of statin prescription was noteworthy. These drugs had been prescribed only to CKD patients. It happened that each patient's prescription status of these drugs was not altered during the study period.

The participants who did not have CKD were those who had wishes to continue follow-up visits to nephrology clinic because of their concern for the disease, typically following suspicious findings in health checkups. These individuals were later determined not to have CKD and were without medication. Among these people, there were three who had borderline hypertension in clinical settings as is shown in Table 2 but were clinically deemed not hypertensive based on findings including home blood pressures. These non-CKD individuals were without medication, which may have contributed to the tendency of slightly longer treatment periods in this study in comparison to CKD patients.

All the participants were generally cooperative toward the study treatment and there were no dropouts or missing data. Judging from conversations at outpatient clinic visits, there were no signs or remarks that suggested inadequate adherence to treatment instructions though we did not take any steps to inspect or count unused tablets.

**Figure 1:**
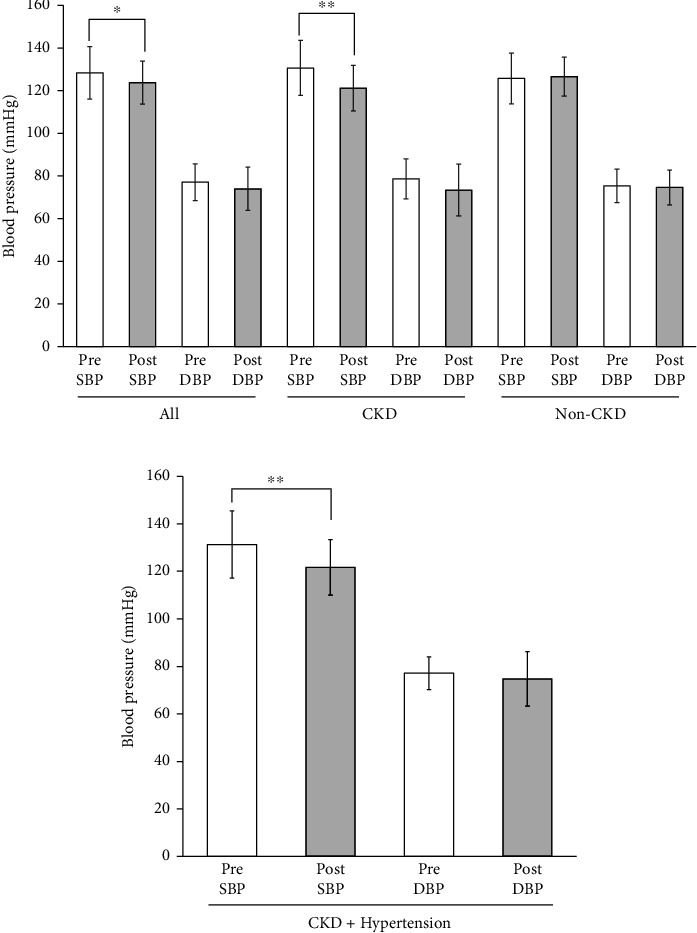
(a) Blood pressure at the start (pre) and at the end (post) of Tochu leaf extract tablet administration according to the CKD status. Values of all participants, of those with CKD and of those without CKD (non-CKD), are shown. (b) Blood pressure at the start (pre) and at the end (post) of Tochu leaf extract tablet administration in CKD patients with hypertension (*n* = 7). SBP: systolic blood pressure, DBP: diastolic blood pressure. ^∗^*p* < 0.05 and ^∗∗^*p* < 0.01.

Mean SBP of all participants decreased from 128.3 ± 12.3 mmHg at the start to 123.8 ± 10.2 mmHg at the end of the administration of Tochu leaf extract tablet (*p* < 0.05) (Figure 1(a)). DBP did not decrease significantly. In participants with CKD alone, SBP further decreased from 130.7 ± 12.9 mmHg to 121.2 ± 10.7 mmHg (*p* < 0.01).

Among these nine CKD patients, seven had hypertension as defined either by having BP levels equivalent to hypertension (SBP ≧ 130 or DBP ≧ 80 at baseline) or by being prescribed antihypertensive medications. To delineate the sensitivity of this blood pressure-lowering effect in relation to the presence or absence of hypertension, we examined the changes in seven hypertensive CKD patients only. Mean SBP was significantly reduced from 131.3 ± 14.1 to 121.7 ± 11.7 mmHg by the treatment (*p* < 0.01), a change comparable to that in all the CKD patients (Figure 1(b)). Mean DBP was reduced nonsignificantly. No significant decrease in blood pressure, either SBP or DBP, was observed in participants without CKD. Changes in BP values of each participant are shown in Table 2.

**Figure 2:**
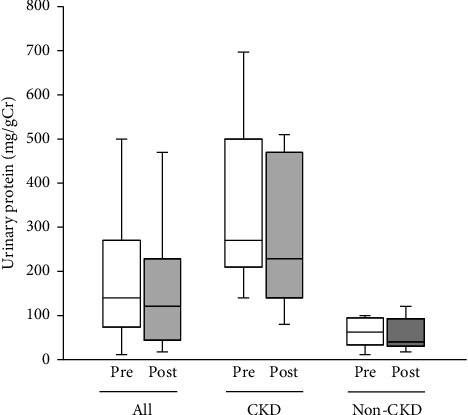
Proteinuria levels at the start (pre) and at the end (post) of Tochu leaf extract tablet administration according to the CKD status. Values of all participants, of those with CKD and of those without CKD (non-CKD), are shown.

**Figure 3:**
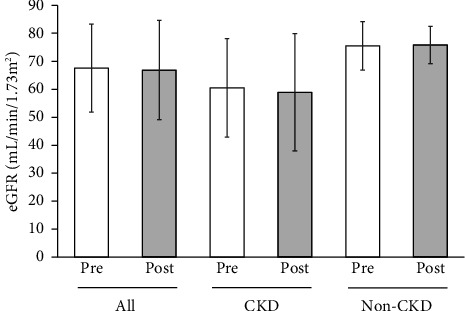
Levels of eGFR at the start (pre) and at the end (post) of Tochu leaf extract tablet administration according to the CKD status. Values of all participants, of those with CKD and of those without CKD (non-CKD), are shown.

No changes were observed in the median urinary protein or mean eGFR of all the participants, of CKD participants alone or non-CKD participants alone (Figures 2 and 3).

**Figure 4:**
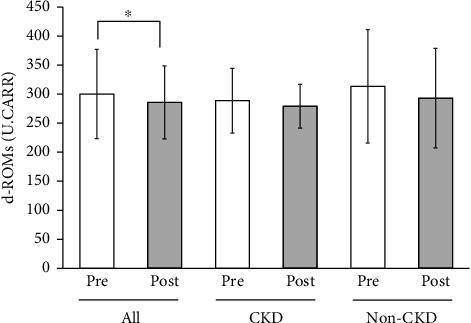
Blood oxidative stress index (d-ROMs) levels at the start (pre) and at the end (post) of Tochu leaf extract tablet administration according to the CKD status. Values of all participants, of those with CKD and of those without CKD (non-CKD), are shown. ^∗^*p* < 0.05.

**Figure 5:**
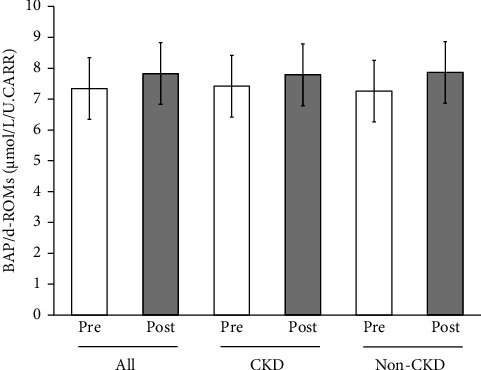
Biological antioxidant potential (BAP) levels divided by d-ROMs at the start (pre) and at the end (post) of Tochu leaf extract tablet administration according to the CKD status. Values of all participants, of those with CKD and of those without CKD (non-CKD), are shown.

Mean blood oxidative stress index (d-ROMs) of all the participants significantly decreased from 300.2 ± 76.7 U.CARR to 285.9 ± 63.0 U.CARR (*p* < 0.05), while there were tendencies of nonsignificant decreases in CKD participants alone or in non-CKD participants alone (Figure 4). There were no significant changes in mean antioxidant capacity (BAP) of all the participants, CKD participants alone or non-CKD participants alone (data not shown). However, when this was analyzed as BAP/d-ROMs ratio, the mean value of all the participants was increased with a *p* value close to statistical significance (*p*=0.05) (Figure 5).

**Figure 6:**
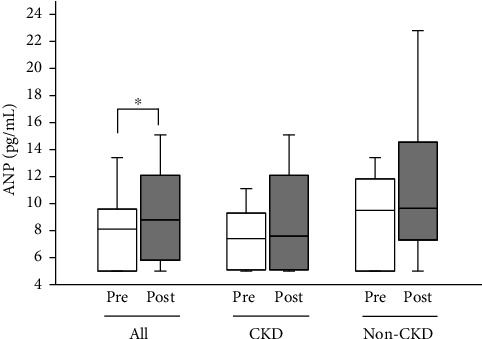
Blood ANP levels at the start (pre) and at the end (post) of Tochu leaf extract tablet administration according to the CKD status. Values of all participants, of those with CKD and of those without CKD (non-CKD), are shown. ^∗^*p* < 0.05.

Median blood ANP of all the participants was increased from 8.1 (5.0–9.6) pg/mL to 8.8 (5.8–12.1) pg/mL (*p* < 0.05), while it tended to increase nonsignificantly in CKD participants alone and in non-CKD participants alone (Figure 6).

As for metabolic, inflammatory, or vascular function indices, no significant changes were observed in HbA1c, lipids (total cholesterol, triglyceride, and HDL cholesterol), adiponectin, MDA-LDH, CRP, or EPC between the two time points (Table 3).

During the study period, there were no serious adverse events which resulted in death or hospital admission. Based on reviewing of the medical charts, there were no symptoms or changes in laboratory data which could be regarded as events of adverse clinical significance.

## 4. Discussion

In this exploratory single-arm clinical study, we investigated effects of relatively short-term Tochu leaf extract administration on blood pressure or other markers such as those related to inflammation or vascular function in participants including CKD patients. As main new findings, significant decrease was observed in the mean SBP of CKD patients, while the difference was not significant in non-CKD patients. In the analysis of all the participants, oxidative stress marker was decreased and ANP was increased.

Though the treatment periods were slightly shorter with CKD patients in comparison to non-CKD individuals in this study, blood pressure-lowering effect by Tochu was more noted in CKD patients. This may be explained, to considerable degrees, by the fact that in this study, only 7 out of 17 participants had hypertension, all of whom also had CKD. If Tochu's effect were analyzed based on hypertension status alone, regardless of CKD, a stronger effect in the hypertensive group would be expected. The absence of hypertensive patients in the non-CKD group limits the ability to compare the antihypertensive effects of Tochu between CKD and non-CKD patients.

Effects of Tochu on redox status demonstrated here are in agreement with previous reports including our own based on DS rats with hypertensive renal damage, which suggested involvement of ROS in the blood pressure modulation by Tochu and GEA [17]. Our current clinical study results will provide further evidence on the mechanism of beneficial effects conferred by Tochu, especially in the context of CKD, through the improvement of oxidative state, which has been widely shown to be involved in blood pressure modulation [20].

ANP in general primarily is known to possess natriuretic and vasodilating properties, exerting beneficial negative feedback to excessive fluid volume or elevated blood pressure [21]. However, ANP reportedly dilates afferent arterioles while constricting efferent arterioles, increasing glomerular capillary pressure [22]. ANP may also promote macrophage secretion of IL-6 and TNF-α via the guanylate cyclase-coupled A receptor [23]. It has been reported that microalbuminuria induced by ANP may contribute to faster kidney function deterioration [24]. In heart failure, an angiotensin receptor-neprilysin inhibitor (ARNI) has in recent years been established as an important component in the therapeutic strategy. ANP can be mechanistically increased by ARNI since this drug reduces ANP degradation by inhibiting neprilysin. ANP was in fact increased by ARNI treatment in heart failure patients, and these increases are reported to be positively associated with the degrees of left ventricular remodeling improvement [25], although in another study, there was a negative association [26]. ANP upregulation elicited by Tochu leaf extract administration, as demonstrated in our study, may be directly beneficial, but this may depend on clinical contexts, requiring further clarification.

As to the mechanism of ANP upregulation by Tochu, the findings in our study may be at least partially in agreement with the mechanism suggested in the work by Kim, where GLP-1R agonist liraglutide reduced blood pressure in mice by inducing ANP secretion in cardiac atia in a manner dependent on this receptor [13]. Gong has found that the iridoids such as GEA are novel orthosteric agonists of rat and human GLP-1Rs that possibly act at the same binding site as exendin 9-39, a specific GLP-1 receptor antagonist, and that GEA functions as a GLP-1R agonist and provides antinociception in chronic pain [27]. Indeed, Nakamura demonstrated that GEA reduces blood pressure in SHR by functioning as a GLP-1R agonist and possibly promoting the release of ANP into peripheral blood from the heart [12].

Upon re-examination of our data, levels of blood pressure reduction were not significantly correlated with ANP increases or with redox status changes (data not shown). This may have been due to the relatively small sample size and short duration of administration, which were the major limitations of this study. Lack of improvement in EPC profile despite previously reported improvements in vascular endothelial function by Tochu [11] may also be due to the small sample size and the short duration of administration. The same reasons may also explain the fact that there were no significant changes in other potentially relevant variables, such as in inflammation or metabolism (oxidized LDL, CRP, adiponectin, HbA1c, or lipids).

As for future directions, further investigations under strengthened study settings with larger numbers of participants and longer treatment periods will be warranted. This may include studies examining effects of Tochu tablet treatment in patients with more advanced CKD stages coupled with conventional drug treatments with ARB or SGLT2 inhibitor. Or alternatively, studies which will examine the effects on patients at borderline stages preceding the apparent onset of diseases like CKD or hypertension may be beneficial since due to its property as food or supplemental product, Tochu extract may be suited for situations where the use of prescribed medicines is not necessarily preferred. However, needless to mention, in every conceivable study situation, it is of utmost importance that close attention has to be paid for the securing of patient safety.

The major limitation of this study, as mentioned above, was that due to the small sample size, it cannot be completely ruled out that the results were due to coincidence. This small number of participants also limits the ability to assess the effects of Tochu across different stages of CKD. Furthermore, since this was a single-arm study without control, it is difficult to completely rule out a possibility that a placebo-like effect arising from study participation may have contributed to the results. However, no instructions were given to the participants to make any particular changes to daily life habits, including diet, other than taking the study supplement. It may well be reasonable to assume that the changes observed were actually caused by Tochu leaf extract intaking.

## 5. Conclusion

In summary, the present single-arm clinical study on participants including mild CKD patients has shown that Tochu leaf extract has antihypertensive effects in CKD patients, as well as presumably overall beneficial effects in blood oxidative stress marker and in ANP levels. These may suggest potential therapeutic promise of Tochu at earlier stages in CKD, which could help to fill the gaps in currently underserved opportunities for prevention or intervention.

## Figures and Tables

**Table 1:** Baseline characteristics of study participants according to the CKD status.

	All (*n* = 17)	CKD (*n* = 9)	Non-CKD (*n* = 8)	*p* value
Age (years)	59.3 ± 16.2	65.2 ± 15.7	52.6 ± 14.9	0.11
Male sex (%)	82.4	88.9	75.0	0.45
Body mass index (kg/m^2^)	24.7 ± 4.5	25.4 ± 4.8	23.9 ± 3.6	0.51
Serum creatinine (mg/dL)	0.91 ± 0.22	1.01 ± 0.24	0.80 ± 0.12	< 0.05
eGFR, mL/min/1.73 m^2^	67.6 ± 15.7	60.5 ± 17.6	75.5 ± 8.6	< 0.05
Urinary protein (mg/gCr)	140 (74–270)	270 (210–500)	62 (34–95)	< 0.001
SBP (mmHg)	128.3 ± 12.3	130.7 ± 12.9	125.8 ± 11.9	0.21
DBP (mmHg)	77.1 ± 8.6	78.7 ± 9.3	75.4 ± 7.9	0.22
Administration period (days)	33 (28–35)	28 (28–28)	35 (35–35)	< 0.001
Drug, cases				
CCB	5	5	0	
ARB	6	6	0	
Thiazide	1	1	0	
Beta-blocker	2	2	0	
MRA	2	2	0	
Statin	5	5	0	
Ezetimibe	1	1	0	
SGLT2 inhibitor	2	2	0	
DPP4 inhibitor	2	2	0	
Xanthine oxidase inhibitor	3	3	0	
Antiplatelet	2	2	0	

*Note:* SGLT2, sodium-glucose cotransporter 2.

Abbreviations: ARB, angiotensin receptor blocker; CCB, calcium channel blocker; CGN, chronic glomerulonephritis; DPP4, dipeptidyl peptidase-4; MRA, mineralocorticoid receptor antagonist.

**Table 2:** BP values and characteristics of each participant.

Age	Sex	CKD	eGFR category	Proteinuria category	Diseases	Antihypertensive drugs	Pre SBP	Post SBP	Pre DBP	Post DBP
60s	M	+	G2	A2	CGN, DM, HTN	MRA	148	137	84	67
70s	M	+	G3a	A3	HTN, DL	ARB, CCB	126	112	74	58
70s	M	+	G3b	A3	Interstitial nephritis, DM, HTN	ARB, CCB, beta-blocker	138	124	63	66
60s	M	+	G3b	A2	CGN, HTN	ARB	127	126	79	84
70s	M	+	G3b	A1	CKD (unknown etiology)	—	121	113	71	56
20s	F	+	G1	A2	CGN	—	136	126	97	82
70s	M	+	G3a	A2	HTN, DM, CAD	ARB, CCB, MRA	147	126	81	84
70s	M	+	G2	A2	HTN, DL	ARB, CCB	108	101	80	89
50s	M	+	G3a	A3	CGN, HTN, hyperuricemia, DL	ARB, CCB, thiazide, beta-blocker	125	126	79	75
50s	M	—	G2	A1	—	—	104	109	67	64
60s	M	—	G2	A1	—	—	123	131	81	82
30s	M	—	G1	A1	White-coat HTN	—	138	124	78	72
50s	M	—	G2	A1	—	—	125	129	85	83
20s	M	—	G2	A1	—	—	118	120	72	76
60s	M	—	G2	A1	—	—	124	127	62	62
60s	F	—	G2	A1	White-coat HTN	—	132	135	76	82
60s	F	—	G2	A1	White-coat HTN	—	142	138	82	76

*Note:* HTN, hypertension; DL, dyslipidemia.

Abbreviations: ARB, angiotensin receptor blocker; CAD, coronary artery disease; CCB, calcium channel blocker; CGN, chronic glomerulonephritis; DM, diabetes mellitus; MRA, mineralocorticoid receptor antagonist.

**Table 3:** Metabolic, inflammatory, and vascular function profiles at the start (pre) and at the end (post) of administration period.

		ALL	CKD	Non-CKD
HbA1c (%)	Pre	5.81 ± 0.38	6.03 ± 0.32	5.52 ± 0.25
	Post	5.78 ± 0.48	6.07 ± 0.47	5.46 ± 0.23
	*p* value	0.59	0.73	0.09

Total cholesterol (mg/dL)	Pre	186 ± 38	178 ± 30	195 ± 45
	Post	191 ± 34	179 ± 26	204 ± 39
	*p* value	0.35	0.92	0.32

Triglyceride (mg/dL)	Pre	122 ± 71	130 ± 82	113 ± 62
	Post	121 ± 55	115 ± 61	129 ± 50
	*p* value	0.995	0.66	0.39

HDL-cholesterol (mg/dL)	Pre	57.0 ± 16.7	54.2 ± 16.1	60.0 ± 18.0
	Post	56.9 ± 17.1	54.3 ± 17.5	59.9 ± 17.4
	*p* value	0.96	0.99	0.95

Adiponectin (μg/mL)	Pre	9.57 ± 3.73	11.30 ± 3.44	7.60 ± 3.14
	Post	9.25 ± 3.53	10.86 ± 3.41	7.44 ± 2.84
	*p* value	0.26	0.34	0.59

MDA-LDL (U/L)	Pre	98.6 ± 30.5	95.9 ± 24.1	101.8 ± 38.0
	Post	95.5 ± 34.9	92.2 ± 38.8	99.1 ± 32.0
	*p* value	0.61	0.64	0.81

CRP (mg/dL)	Pre	0.074 (0.036–0.222)	0.074 (0.007–0.222)	0.076 (0.037–0.181)
	Post	0.050 (0.034–0.078)	0.050 (0.013–0.22)	0.051 (0.035–0.063)
	*p* value	0.13	0.71	0.08

EPC (%)	Pre	0.102 (0.067–0.126)	0.102 (0.067–0.123)	0.094 (0.066–0.128)
	Post	0.096 (0.072–0.123)	0.077 (0.067–0.093)	0.120 (0.103–0.135)
	*p* value	0.75	0.65	0.95

*Note:* HDL-cholesterol indicates high-density lipoprotein-cholesterol; MDA-LDL, malondialdehyde-modified LDL.

Abbreviations: CRP, C-reactive protein; EPC, endothelial progenitor cell.

## Data Availability

The data that support the findings of this study are available on request from the corresponding author. The data are not publicly available due to privacy or ethical restrictions.
